# Fur Cortisol in French Bulldogs with Different Manifestations of Brachycephalic Obstructive Airway Syndrome

**DOI:** 10.3390/ani14071060

**Published:** 2024-03-30

**Authors:** Maike Schroers, Juliette Goossens, Yury Zablotski, Andrea Meyer-Lindenberg

**Affiliations:** Clinic of Small Animal Surgery and Reproduction, Ludwig-Maximilians-University Munich, 80539 Munich, Germany

**Keywords:** canine, dog, stress, cortisol, fur, BOAS

## Abstract

**Simple Summary:**

Brachycephalic Obstructive Airway Syndrome (BOAS) is a common disease in short-nosed dog breeds associated with narrowing of the airways. The disease is associated with frequent respiratory distress and chronic stress for the animals. In this study, the stress hormone cortisol was measured in the fur of dogs with BOAS and healthy dogs. It was shown that a single measurement of cortisol in the animal’s fur cannot be used to distinguish between dogs clinically affected with BOAS and those without clinically relevant signs of the disease. There was no statistically significant difference between the age of the animals and the fur cortisol. The measurement of hair cortisol to assess chronic stress in brachycephalic dogs remains challenging using a single sample measurement.

**Abstract:**

In the present study, 33 French bulldogs with varying degrees of brachycephalic obstructive airway syndrome (BOAS) were evaluated for various parameters to provide evidence of chronic stress in the dogs. For this purpose, an owner interview, a clinical examination, and cortisol concentrations in the fur of the dogs were collected. The median cortisol concentration in the fur of the dogs suffering from BOAS (n = 19) was 0.99 pg/mg (range 0.63–66.18), while that of the control group (n = 14) was 1.13 pg/mg (range 0.3–43.45). However, the statistical analysis did not reveal a statistically significant difference; therefore, it is not possible to distinguish between dogs clinically affected with BOAS and those without clinically relevant signs of the disease. There was no statistically significant difference between the age of the animals and the fur cortisol. None of the owners in the examination and control groups indicated that their dog was suffering from chronic stress which shows that the disease is easily underestimated and remains common. Thus, a thorough clinical examination by an experienced veterinarian remains the gold standard in order to diagnose BOAS.

## 1. Introduction

Brachycephalic obstructive airway syndrome (BOAS), which frequently occurs in brachycephalic dog breeds such as pugs and French bulldogs, has been described and investigated in numerous studies over many years [1,2,3,4,5,6]. The breeds are considered particularly appealing due to their outwardly “childlike” facial features [7]. However, they often suffer from stenotic nostrils, an overlong soft palate, laryngeal constriction and abnormal chonchal growth due to the unphysiological proportions of the skull and soft tissue, which can lead to breathing problems and, in severe cases, even syncope and collapse [1,2,8,9,10]. The affected animals exhibit respiratory sounds even at rest, which can serve as a subjective measure of airway obstruction or narrowing. Roedler et al. (2013) [5] were able to show that mesocephalic dog breeds do not exhibit respiratory sounds at rest and that these should therefore always be regarded as pathological. During sleep, brachycephalic breeds also have an increased risk of sleep disorders, as Amis and Kurpershoek (1986) [11] were able to demonstrate in a study comparing the breathing patterns of brachycephalic and non-brachycephalic dogs. In a further study, in which owners of brachycephalic dogs were asked about their quality of life, it was confirmed that the animals had sleep disorders in addition to limited physical activity and sensitivity to heat [2,5,12]. Despite the known predisposition to diseases in these breeds, the number of pets with brachycephalic breeds is steadily increasing [6,13]. Many owners often do not perceive the complaints as a disease, but as “typical for the breed” [14]. The habituation to the breathing sounds of the animals can lead to the chronic stress of the animals being underestimated [14,15]. 

Chronic stress can be assessed in several ways. In human medicine, for example, specially designed questionnaires can be used for this purpose, which are answered by the patients [16]. Furthermore, the physical consequences of chronic stress can be determined, for example, which are associated with the release of inflammatory mediators and other reactions of the immune system in the body [17]. In addition, hormones associated with stress can be detected, for example, in the blood, urine, saliva, or hair [18,19,20,21]. 

The glucocorticoid cortisol, which is controlled by the hypophysis–hypothalamus–adrenal axis (HPA), has already been established in human and veterinary medicine to assess stress [18,22,23]. However, it is noteworthy that in addition to stress, diseases of the HPA axis can also influence the release of cortisol concentrations [22].

While cortisol concentrations in blood [24] and saliva [25], e.g., can be indicative of acute stress, cortisol measurement in hair has gained increasing attention over the past decade in humans and animals for the assessment of chronic stress. In particular, many studies can be found on the assessment of psychological stress, e.g., for shift workers [26] or patients with depression [27,28]. In dogs, studies also exist to measure fur cortisol, such as when considering the stress caused by different housing styles [29] or housing conditions of dogs [30] or the relationship between stress of the dog and its owner [31]. In addition, a study has shown that fur color and nutritional status of a dog can influence the cortisol concentration in the fur [32]. In a study with Border Collies, a correlation between fur cortisol and an epilepsy disorder was also demonstrated [33]. Additionally, factors in everyday life, such as the number of other dogs in the household, can influence chronic stress and thus also the fur cortisol in the border collies investigated [33]. 

Studies on the association between chronic diseases and fur cortisol of brachycephalic dogs do not yet exist in the accessible literature. Therefore, the present pilot study aimed to investigate cortisol and concentrations in the fur of French bulldogs using liquid chromatography-tandem mass spectrometry (LC-MS/MS) [34] in order to verify whether the measured hormone concentrations in the fur can be used to differentiate between dogs clinically affected with BOAS and those without clinically relevant signs of the disease.

## 2. Materials and Methods

### 2.1. Animals

All studies were approved by the Ethics Committee of the Veterinary Faculty, Ludwig-Maximilians-University Munich, Germany (Reference 167-02-05-2019). The study group included French bulldogs that were presented to the Clinic of Small Animal Surgery and Reproduction, Ludwig-Maximilians-University, Munich, Germany, due to moderate (grade 2) or high-grade (grade 3) BOAS primarily for treatment of their BOAS. The control group had no (grade 0) or low-grade BOAS (grade 1) whose owners voluntarily participated in the study and who were presented to the clinic for other conditions (e.g., neutering). Dogs with grade 0 or grade 1 were controls and dogs with grade 2 or grade 3 were assigned to the study group. Exclusion criteria were body condition score (BCS) below 4/9 and above 5/9 as obesity and cachexia can also have an influence on cortisol metabolism. Furthermore, dogs with acute orthopedic and internal diseases up to six months prior to study participation (e.g., cruciate ligament rupture or discopathy); chronic orthopedic and internal diseases associated with stress and pain for the animals; and pituitary, hypothalamic or adrenal gland diseases were excluded. In addition, the owners had to confirm that they had not administered topical or systemic cortisone to their dogs in the last 6 months. 

The degree of BOAS was assessed by a thorough clinical examination according to the grading of Liu et al. (2017) by two examiners [35]. A standardized fitness test (3 × 5 min of trotting with 1 min of rest each) was performed on a treadmill [36] and during the breaks as well as after the test, the dogs were evaluated according to the University of Cambridge Respiratory Function Grading Scheme [35]. Overall, the fitness test was carried out on all dogs, with the exception of one dog (BOAS grade 0), which did not accept the treadmill.

### 2.2. Owner Survey

The following life circumstances were asked in the medical history:

- Place of residence (city/country);
- Previous illnesses;
- Change of living conditions in the last 6 months (e.g., moving);
- Children in the household and whether they can be a trigger for stress;
- Partner animals in the household and whether these can be a trigger for stress;
- Loneliness of the animal in everyday life;
- Dog sitter and whether this is a trigger for stress;
- Perception of the owner whether the dog suffers from chronic stress.

### 2.3. Fur Sample Collection

Fur samples were collected from the abdomen in the umbilical region using an electric shearing machine (ISIS, AESCULAP) (area 2 cm × 2 cm) and then wrapped in aluminum foil and stored at room temperature for up to 6 months. After sample collection was completed, they were shipped to DRESDEN LAB SERVICE GMBH of Dresden University of Technology and analyzed by liquid chromatography-tandem mass spectrometry (LC-MS/MS) as described by Gao et al. (2013) [34]. Statistical analysis was performed using a Wilcoxon–Mann–Whitney test (significance level *p* ≤ 0.05).

## 3. Results

A total of 33 French bulldogs were included in the study, including 19 moderate to high grade (grade 2, n = 16; grade 3, n = 3) BOAS-affected dogs (study group) and 14 non to very low-grade (grade 0, n = 4; grade 1; n = 10) BOAS-affected French bulldogs (control group). The study group consisted of eight male and eleven female dogs with an average age being five years (range 1–13) and an average weight being twelve kg (range 6–15). In the control group there were two males and twelve females with an average age being five years (range 1–9) and an average weight being eleven kg (range 8–15).

Overall, 15% (5/33) of the owners indicated that there were children in the household; however, according to the owners, they were not a cause of chronic stress. Similarly, 39% (13/33) of the owners indicated that there were one or more partner animals in the household, although these would also not be a cause of chronic stress. 3% (1/33) of the owners in the study group indicated that the animal was often alone for long periods of time, although he also indicated that this was not associated with chronic stress for the animal. None of the owners stated that the animal was frequently with a dog sitter. In addition, none of the owners considered that their dog suffered from chronic stress, neither in the study group nor in the control group.

Even though the dogs did not suffer from acute pain according to the questionnaires, the owners still reported their dogs’ pre-existing conditions, although these had been successfully treated at least six months previously. Thus, in the study group, three dogs had a history of eye disease, one patient had a patellar luxation, one patient had a cruciate ligament rupture, one patient had a dermoid cyst, one patient had a demodex infestation, and six patients had food allergy, which, however, could be well controlled with the help of adapted feeding. In the control group, two patients had a herniated disc in the past, one patient had a patellar luxation, one patient had a benign mammary tumor, one patient had a nictitating membrane injury, and one patient also had an allergy that could be well adjusted.

**Figure 1:**
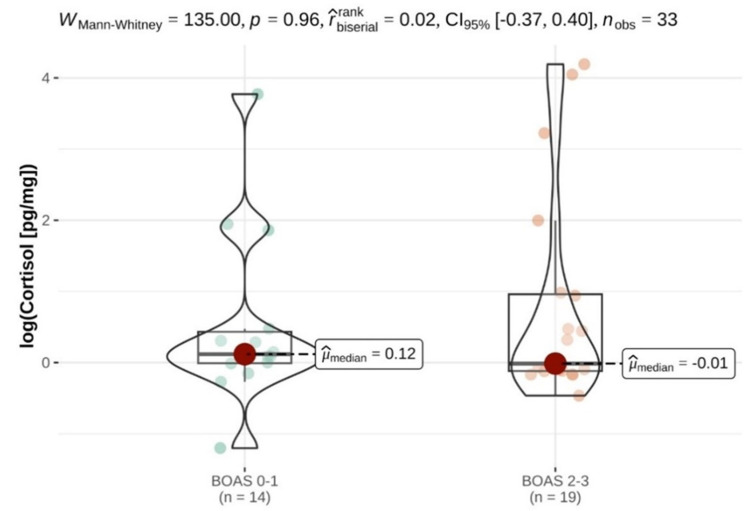
Logarithm of cortisol concentrations in fur to show the difference between control (BOAS 0–1) and control (BOAS 2–3) groups; µ median = mean value.

The median cortisol concentration in the fur of the dogs suffering from BOAS (n = 19) was 0.99 pg/mg (range 0.63–66.18), while that of the control group (n = 14) was 1.13 pg/mg (range 0.3–43.45) (Table 1), but the statistical analysis did not show any statistical significance, so it was not possible to distinguish between the study and control groups based on cortisol concentrations alone (Figure 1).

Regarding sex, the median cortisol concentration in the fur of the female dogs was 1.0 (0.63–7.01) pg/mg and that of the male dogs was 2.56 pg/mg (range 0.85–66.58). The great difference can be attributed to the four outliers (three of the study group and one of the control group) among the male animals. Overall, the correlation between gender and fur cortisol was not statistically significant (*p* = 0.06). 

**Figure 2:**
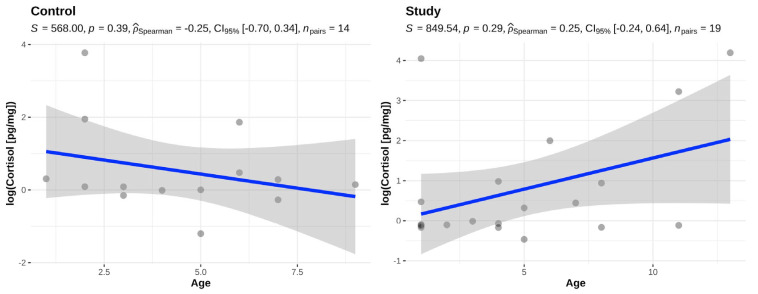
Association between age and fur cortisol in the control (BOAS 0–1) and study group (BOAS 2–3).

There was no statistically significant difference between the age of the animals and the fur cortisol in either the study group (*p* = 0.29) or the control group (*p* = 0.39) (Figure 2). 

Pre-existing conditions that had to be successfully treated at least six months earlier were orthopedic diseases, skin diseases, benign mammary tumors, eye diseases, and allergies, which, however, could be well controlled with the help of adapted feeding. Of the four patients with particularly high cortisol concentrations, one dog in the control group (patient 13) had previously reported patellar luxation, one patient in the study group (patient 10) had no other previous diseases, and two patients (patients 9 and 12) had food allergies. Overall, no significant deviations from the average cortisol concentrations were seen in the patients with allergies. 

The majority of the dogs in the present study had a light coat color in the umbilical region. In the study group, this was white in 15 dogs, white-gray in two dogs, brown in one dog, and black in one dog (Table 1). In the control group, the coat color was white in eight dogs, white-gray in one dog, black in three dogs, black-white in two dogs, and black-white-brown in one dog (Table 1).

## 4. Discussion

The aim of the current study was to determine whether cortisol levels in the fur may be used to indicate chronic stress in brachycephalic dogs. We therefore investigated whether a one-time measurement of cortisol in the fur of French bulldogs can be used to distinguish between dogs with and without clinically significant BOAS. 

It was shown that the cortisol measurement in the fur of brachycephalic dogs was not helpful for assessing chronic stress in these dogs. The results are contrary to the study by Packer et al. (2019) [33], in which a positive correlation between fur cortisol and epilepsy, as well as chronic stress factors in everyday life, was found in Border Collies. In addition to the higher number of animals, a possible reason for the different results could be that healthy Border Collies and Border Collies with epilepsy were included in the study. The present study lacks such a fully healthy control group. The high prevalence of BOAS makes it difficult to find brachycephalic dogs that are completely healthy and show no signs of BOAS. The authors therefore agreed to define the control group as dogs with no or very few signs of BOAS. Larger studies with a higher number of dogs showing no signs of BOAS at all would be useful to better assess the impact of BOAS on chronic stress in dogs especially since the risk of a type 2 statistical error increases with a small number of samples. 

It should be considered that the grading of the BOAS is always associated with a subjective component, so that there may also be overlaps between the groups. It is therefore conceivable that all brachycephalic dogs, even those with only very mild signs of BOAS, suffer from chronic stress. This could explain the lack of difference between the study and control groups and could be clarified in a follow-up study examining the fur cortisol of brachycephalic and non-brachycephalic dogs. 

Despite the contradictory results with the study by Packer et al. (2019) [33], a correlation between chronic stress and a single hair sample was not always found in human medical studies either. Here, again, there are contradictory results among the studies. In a study by Braig et al. (2016) [27], cortisol was measured in the hair of women who suffered from chronic stress and depression after childbirth and compared with the values of women who suffered from little stress; no correlation between hair cortisol and chronic stress was detectable here either. This is in contrast to another human medical study, in which a single hair sample for measuring cortisol was used to distinguish between patients with depression and a mentally stable control group. Here, it could be shown that the cortisol levels in the hair of patients with depression were significantly higher [37]. Additionally, another human medical study showed that in patients with chronic stress, the HPA axis “dulls” and less cortisol is released overall [38]. Cortisol concentrations in the body thus appear to depend on the nature of the stress and pain, as well as the intensity and duration, and need to be examined and assessed on an individual basis [39]. The French bulldogs in the present study also suffered from varying degrees of BOAS and thus stress, which is probably why the HPA axis is affected to different degrees in the dogs, making it difficult to compare the results of the hormones in the fur. In dogs suffering from BOAS, it can be assumed that the animals suffer from chronic stress due to the permanent constriction of the airways [8,13,35,40]. However, the function of the HPA axis was not further investigated in the present study and therefore cannot be assessed. For this purpose, a cortisol stimulation test would have to be performed, i.e., cortisol concentrations in blood or saliva would have to be examined before and after a standardized stress situation. Regarding the dog, there are two studies so far in which cortisol was not measured once in the fur, but the course of cortisol fluctuations in the context of a stress situation was assessed, e.g., in a study in which the housing conditions of dogs were changed [29,30]. Due to the individual variations of cortisol concentrations in the fur, several measurements before and after a stress situation may seem to be more useful than a single fur sample examination. 

In addition, a stress or pain situation should always be assessed using different examination methods, such as a clinical examination and a patient or patient owner interview. Hence, in the present study, the clinical examination easily identified the patients with moderate (grade 2) and high-grade (grade 3) BOAS. In contrast, in the owner survey, none of the owners, even of the dogs severely affected by BOAS, considered that their pet was suffering from chronic stress. This illustrates the subjective perception of the BOAS. The results are also consistent with previous owner surveys in America, England [41], Canada [42] and Germany [14], which have shown that BOAS in brachycephalic dog breeds is often not judged as a “disease” but as “typical for the breed”, thus significantly underestimating the suffering of the dogs due to the respiratory problems associated with the syndrome. In addition, it is possible that the environmental factors examined in this study are actually causing the animals stress without the awareness of the owners. 

Cortisol concentrations were measured by LC-MS/MS in the present study because it was available to the authors, but it can also be measured by ELISA as in the other studies [30,32]. Different methodologies may be the reason why studies have different results. Although there are no previous data on hormone concentrations in the fur of French bulldogs, the cortisol concentrations in the present study were significantly lower than those in previous studies on dogs of other breeds. Thus, the average concentrations were 9.17 pg/mg (range 0.63–66.18) in the study group and 4.89 pg/mg (range 0.3–43.45) in the control group. Other studies did not include specific dog breeds, which may explain possible differences. For example, the average cortisol concentrations in the fur of healthy dogs in the study on possible factors influencing fur cortisol were 17.87 pg/mg (range 6.1–121.2) [32]. In another study, in which the cortisol concentrations in the fur of healthy dogs were measured before and after adoption of the dogs, the average concentrations in the study group were 16.0 ± 6.8 pg/mg and in the control group 17.9 ± 7.1 pg/mg, i.e., also higher [30]. However, the low concentrations seem to be mainly due to the test methodology, as the concentrations measured by LC-MS/MS are often significantly lower than those measured by Enzyme-Linked Immunosorbent Assay (ELISA) [43]. 

In the present study, fur was collected using the same clippers and from the same body site in the umbilical region to keep the study design as consistent as possible. Overall, the majority of dogs in the present study had white fur color in the umbilical region. However, the cortisol concentrations of the dogs with brown, gray, or black coat color did not differ from the average cortisol concentrations of all dogs. Another study was able to show that color may very well have an influence on cortisol concentrations [32]. In the study on stress in Border Collies with and without epilepsy, only white fur samples from the neck region were also selected [33]. 

Another factor that may have influenced the results of the present was a lack of standardized housing conditions for the dogs before sampling, which is not possible in a clinical study. Thus, environmental factors or even the stress of the owners and the relationship between dog and owner may have an influence on the stress level of the dogs [44]. The fur samples in the study by Packer et al. (2019) [33] were taken by the owner in their usual environment. The extent to which short-term stress on the day of sampling influences the results would need to be clarified in a comparative study. 

In terms of methodology, it is also possible to measure cortisol in blood, saliva, and urine [45,46,47]. While the course of cortisol in the context of an acute stress situation in dogs can be traced in blood [45] and saliva [47], cortisol in saliva and urine [46] can also provide indications of chronic stress. However, the responsiveness of the HPA axis is primarily tested here by measuring cortisol concentrations over a certain period of a stressful situation. This would also be conceivable in a follow-up study with brachycephalic dog breeds.

The average age of the dogs in the present study was five years in both the study and control groups. Bowland et al. (2020) [32] were able to show in their study that age does not seem to have any influence on the cortisol concentrations in the fur, meaning that this factor is probably also negligible in the present study. The average age of the Border Collies in the study by Packer et al. (2022) [33] was also 5 years, although the age range was slightly lower (±2.7 years) than in the present study. Cortisol levels were not associated with age in the present study. It can be assumed that with increasing age and worsening of the BOAS, the chronic stress of the dogs increases. However, cortisol concentrations in the fur could not reflect this. Accordingly, the study by Packer et al. (2019) [33] also found a correlation between the number of previous epileptic seizures and the cortisol level.

There was no significant difference in the fur cortisol between male and female animals; the three neutered dogs were not included in the comparison. Overall, the female animals were clearly overrepresented with 11/33. Nonetheless, Packet et al. (2022) also found no correlation between fur cortisol and sex. In a human medicine study in which the relationship between hair cortisol and the stress level of medical students (n = 74) was investigated, the proportion of women was 56% and the authors described no direct influence of gender on hair cortisol [28]. 

One dog (grade 0) did not complete a performance test. As it did not show any symptoms of BOAS at clinical examination, it was still included in the control group, as it should have shown symptoms at clinical examination in the case of grade 2 and 3 BOAS.

Even though the dogs did not suffer from acute pain according to the questionnaires, the owners of both the study and control groups reported their dogs’ pre-existing conditions, which had been successfully treated at least six months previously, including orthopedic conditions, skin conditions, benign mammary tumors, ocular conditions, and allergies, which could be well controlled with adapted feeding. Previous studies have shown that many brachycephalic dog breeds are predisposed to comorbidities such as ocular, gastrointestinal, spinal, and skin diseases (usually due to allergy) [1,2,8,35,40]. Therefore, patients with pre-existing conditions were not all excluded in the present study, provided that they were successfully treated and thus not associated with chronic stress. The focus of the study was BOAS, which unfortunately often cannot be fully treated and was not treated in the present study, which is why it is associated with permanent stress and suffering in affected patients. A thorough clinical examination by an experienced veterinarian remains the gold standard for evaluating BOAS in order to diagnose the disease. As the disease remains common and is easily underestimated, ways must be found to avoid breeding with dogs affected by BOAS.

## 5. Conclusions

The present pilot study of French bulldogs did not find an association between fur cortisol and the severity of BOAS. A larger follow-up study comparing fur cortisol in brachycephalic and non-brachycephalic dogs could clarify whether dogs of a brachycephalic breed are all subject to chronic stress or whether there are differences within the breed and to other breeds. None of the owners in the examination and control groups indicated that their dog was suffering from chronic stress. Thus, a thorough clinical examination by an experienced veterinarian remains the gold standard in order to diagnose BOAS. 

## Figures and Tables

**Table 1:** Cortisol concentrations in the fur of French bulldogs with different degrees of brachycephalic obstructive airway syndrome (BOAS); study group: BOAS grade 2–3 (right), control group: BOAS grade 0–1 (left).

Patient No	BOAS Grade	Cortisol (pg/mg)
8	0	0.30
15	0	1.36
26	0	0.99
29	0	1.61
6	1	6.43
13	1	43.45
16	1	7.01
19	1	1.33
20	1	0.76
21	1	1.00
22	1	0.86
23	1	1.09
24	1	1.09
31	1	1.16
1	2	0.63
2	2	0.85
3	2	0.91
4	2	0.85
5	2	0.89
7	2	1.60
9	2	66.18
10	2	57.26
11	2	7.37
12	2	25.11
14	2	1.56
18	2	2.56
25	2	0.84
27	2	2.67
28	2	1.38
30	2	0.93
17	3	0.99
32	3	0.90
33	3	0.89

## Data Availability

The data are not publicly available due to privacy and confidentiality.
